# Characterization of grating monochromator performance based on start-to-end FEL pulse propagation method

**DOI:** 10.1107/S1600577525006472

**Published:** 2025-07-31

**Authors:** Ye Zhu, Chuan Yang, Kai Hu, Chen Wu, Zhenjiang Xing, Zhongmin Xu, Weiqing Zhang

**Affiliations:** a Institute of Advanced Light Source Facilities, Shenzhen518107, People’s Republic of China; b Southern University of Science and Technology Shenzhen Guangdong518055 People’s Republic of China; c National Synchrotron Radiation Laboratory University of Science and Technology of China Hefei Anhui230029 People’s Republic of China; d State Key Laboratory of Chemical Reaction Dynamics, Dalian Institute of Chemical Physics Chinese Academy of Sciences Dalian116023 People’s Republic of China; Bhabha Atomic Research Centre, India

**Keywords:** beamline design, grating monochromator, pulse propagation, free-electron laser, synchrotron radiation

## Abstract

Grating monochromators are crucial optical elements in soft X-ray free-electron laser (XFEL) beamlines. Accurately evaluating the properties of grating monochromators with near-realistic XFEL pulse is of paramount importance. In this study, we utilize the start-to-end pulse propagation method to conduct a characterization of grating monochromator performance at the FEL-1 beamline of S^3^FEL. The primary focuses include evaluating the monochromator’s resolving power, assessing the impact of longitudinal source jitter on resolving power, analyzing diffraction effects due to grating’s limited aperture and investigating the influence of thermal deformation. The novelty of this research lies in the direct use of FEL pulse propagation, providing simulation results that are more reliable than those obtained using Gaussian sources. The simulations reveal that the resolving power of the monochromator is significantly influenced by the aforementioned factors, highlighting the importance of considering real FEL beam characteristics in the design and evaluation of grating monochromators for FEL applications.

## Abstract 2

A start-to-end FEL pulse propagation method has been developed to evaluate grating monochromator performance, quantifying the impacts of longitudinal source jitter, finite-aperture diffraction, and thermal deformation on resolving power, thereby offering realistic guidance for FEL beamline design and optimization.

## 1. Introduction

In recent years, X-ray free-electron lasers (XFELs) have undergone rapid development. XFELs can provide ultrashort X-ray pulses with unprecedented high coherence and high peak brightness, enabling ultrafast dynamics studies at the molecular and atomic levels (Young *et al.*, 2010 ▸; Young *et al.*, 2018 ▸; Pandey *et al.*, 2020 ▸). This capability is crucial for scientists to explore the microscopic world, discover new scientific laws and achieve technological breakthroughs. Currently, the XFEL facilities that are in operation or under construction around the world include LCLS (Emma *et al.*, 2010 ▸), SACLA (Ishikawa *et al.*, 2012 ▸), PAL-XFEL (Kang *et al.*, 2017 ▸), SwissFEL (Milne *et al.*, 2017 ▸), European XFEL (Decking *et al.*, 2020 ▸), FLASH (Ayvazyan *et al.*, 2006 ▸) and SHINE (Zhao *et al.*, 2018 ▸). Each of these facilities includes the soft X-ray regime, where grating monochromators are often employed to monochromatize the FEL pulses. In the design and optimization of a grating monochromator, numerical evaluation of its optical performance is an essential step.

In the X-ray beamline design community, many software packages based on either geometrical ray-tracing or wavefront propagation have been employed to evaluate the performance of grating monochromators. Ray-tracing codes include the widely used *SHADOW* (Welnak *et al.*, 1994 ▸), as well as *RAY* (Feldhaus, 1984 ▸) and *XRT* (Klementiev & Chernikov, 2014 ▸). Wavefront propagation tools encompass *SRW* (Chubar & Elleaume, 1998 ▸; Chubar *et al.*, 2008 ▸), *PHASE* (Bahrdt, 1997 ▸; Bahrdt & Flechsig, 1997 ▸), *MOI* (Meng *et al.*, 2015 ▸), *WPG* (Samoylova *et al.*, 2016 ▸) and *WISER* (Manfredda *et al.*, 2022 ▸). Additionally, packages such as *HYBRID* (Shi *et al.*, 2014 ▸) extend ray-tracing by incorporating wavefront propagation corrections, thereby enabling accurate simulation of diffraction effects. In general, beamline scientists assess the resolving power by introducing two Gaussian beams with a wavelength difference of Δλ, allowing them to pass through the monochromator and then applying the Rayleigh criterion to determine if the two beams with different wavelengths can be distinctly resolved at the exit slit. However, there is still a lack of more realistic simulation methods to characterize the performance of grating monochromators.

In our previous work, we developed a software called *FURION* (Zhu *et al.*, 2024 ▸; Hu *et al.*, 2025 ▸), which is capable of simulating the propagation of three-dimensional (3-D) FEL pulses, *E*(*x*, *y*, *t*), in dispersive beamline systems. This start-to-end simulation has already been employed to accurately evaluate the pulse duration, intensity distribution in the time domain, pulse front tilt and pulse front rotation after an X-ray pulse diffracted by a concave VLS grating. This approach can also be applied to simulate chirped pulse compression by a grating pair compressor. In this paper, we develop a method to characterize the performance of grating monochromators based on this simulation. This paper is organized as follows. In Section 2, we briefly review the method for ultra-short pulses diffracted by variable-line-spacing (VLS) gratings. In Section 3, taking the FEL-1 beamline of S^3^FEL as a case study, we present a start-to-end simulation method to evaluate the resolving power of the grating monochromator. In Sections 4, 5 and 6 we utilize this method to study effects of longitudinal source jitter, the diffraction of the grating’s limited aperture and thermal deformation on the resolving power, respectively.

## 2. Pulse diffracted by VLS grating

In this section, we briefly review the methods for ultra-short X-ray pulses diffracted by VLS gratings. In our previous work, we established six-dimensional (6-D) Kostenbauder matrices for different types of X-ray optics (Hu *et al.*, 2023*a* ▸), where a 6-D vector **V** = (*x*, θ_*x*_, *y*, θ_*y*_, *t*, ω) is employed to characterize an ultra-short pulse. Here, *x* and *y* are the transverse coordinates, θ_*x*_ and θ_*y*_ denote the divergences, and *t* and ω refer to time and angular frequency. Then we developed an approach for 3-D ultra-short X-ray pulses propagating in a plane VLS grating monochromator beamline (Zhu *et al.*, 2024 ▸; Hu *et al.*, 2023*b* ▸; Xing *et al.*, 2025 ▸).

The toroidal VLS grating serves as a unified model for gratings. According to the 6-D Kostenbauder matrix for a toroidal VLS grating, the focal lengths in the sagittal (*x*) and meridional (*y*) dimensions can be expressed as 
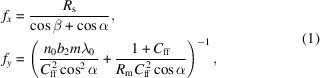
where *R*_s_ and *R*_m_ represent the radii of curvature in the sagittal and meridional dimensions, respectively, α and β are the incident and diffraction angles, respectively, *C*_ff_ = 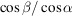 denotes the asymmetry factor, *n*_0_ is the central line density, *m* is the diffraction order and *b*_2_ is the VLS parameter.

The transverse coordinates (*x*′, *y*′) after the toroidal VLS grating are related to the (*x*, *y*) before the grating and can be expressed as 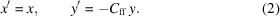
The next step is to achieve the phase modulation of 3-D XFEL pulses by the unified grating model in the (*x*′, *y*′, ω) space, and we have 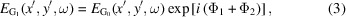
where Φ_1_ corresponds to the phase-modulation induced by the grating’s focusing properties and Φ_2_ corresponds to the phase-modulation due to the dispersive properties of the grating. The unified focusing modulation Φ_1_ can be expressed as 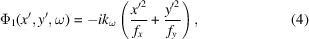
where *k*_ω_ = ω/*c*. The extra phase shift Φ_2_ is regarded as angular dispersion modulation and we have 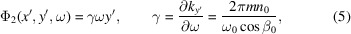
where 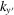 refers to the component of the wavevector in the *y* dimension. Performing inverse Fourier transform on *E*_G1_(*x*′, *y*′, ω), the 3-D XFEL pulse after the VLS grating can be written as 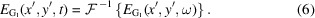
With the *E*_G1_(*x*′, *y*′, ω) obtained after the grating monochromator, we can determine the monochromator’s resolving power and pulse stretching characteristics, as well as evaluate the effects of changes in beamline parameters on its performance.

## 3. Resolving power characterization

In this section, we present a method to characterize the resolving power of grating monochromators based on start-to-end FEL pulse propagation. In the investigation, we take the grating monochromator in the FEL-1 beamline of S^3^FEL as a case for analysis.

**Figure 1:**
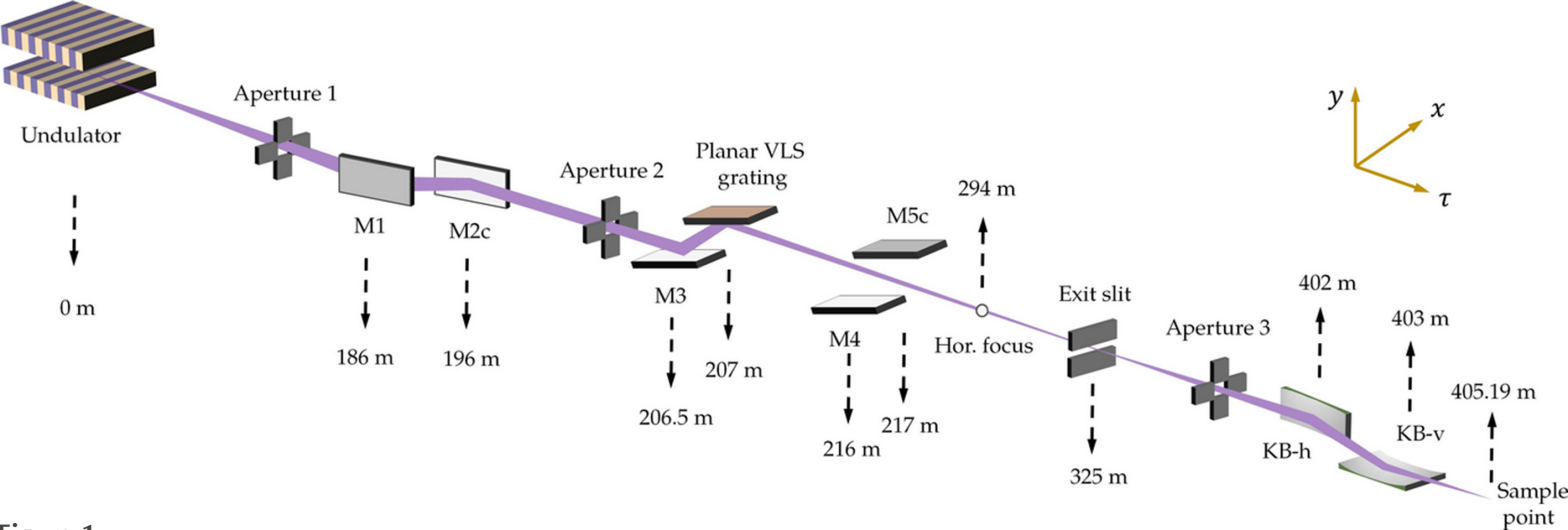
The preliminary optical layout of the SCI branchline of FEL-1 beamline. The locations of the optics from the source are marked by dashed arrows.

The FEL-1 beamline operates in self-amplified spontaneous emission (SASE) mode, with a working photon energy range covering 400–1240 eV. This beamline aims to accommodate three experimental endstations, including the Spectroscopy and Coherent Imaging endstation (SCI), Ambient-Pressure X-ray Photoelectron Spectroscopy endstation (AP-XPS) and Resonant Soft X-ray Scattering endstation (RSS). Fig. 1 ▸ illustrates the preliminary layout of the SCI branchline of FEL-1 beamline. When plane mirror M3 and plane VLS grating are inserted into the beam path and plane mirror M4 and elliptical cylinder mirror M5c are removed, the beamline operates in monochromatic mode. Conversely, when M3 and G are removed from the beam path and M4 and M5c are inserted, the beamline operates in pink mode. This work primarily focuses on the monochromatic mode, in which the pre-mirror (M3) and the plane VLS grating together constitute the grating monochromator. With an object distance of 207 m and an image distance of 118 m, the FEL pulses are focused by the grating onto the exit slit, where different photon energies are spatially separated along the *y* dimension.

The parameters of the plane VLS grating are given in Table 1 ▸. Here, *r*_1_ and *r*_2_ refer to the object distance and image distance of the VLS grating, respectively. The VLS equation is defined as *n*(*w*) = 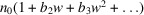. For the case of 1240 eV, *n*_0_ = 300 lines mm^−1^, *C*_ff_ = 1.8, α = 89.062°, β = 88.312°, *b*_2_ = 0.02884 m^−1^ and *R*_m_ = ∞. Substituting these into equation (1) yields a focal length of *f*_*y*_ = 100.288 m for the grating monochromator.

To simulate the ideal resolving power of the monochromator, the 3-D FEL pulse is longitudinally sliced in the (*y*, *t*) domain. This implies that for the *i*th slice we can obtain two-dimensional light field data represented as *E*(*x*_*i*_, *y*, *t*). Using the *FURION* software package, the 3-D FEL pulse sequentially is reflected by M1, M2, M3 and G, finally reaching the exit slit. By observing the spectral distribution of the FEL pulse after the exit slit, the full width at half-maximum (FWHM) of the spectrum is obtained, which is defined as Δ*E*. The resolving power of the monochromator is then calculated using *E*/Δ*E*. Here, the ideal conditions refer to the absence of factors such as source jitter, optical element vibration, surface profile and the strain induced by thermal load and clamping.

**Figure 2:**
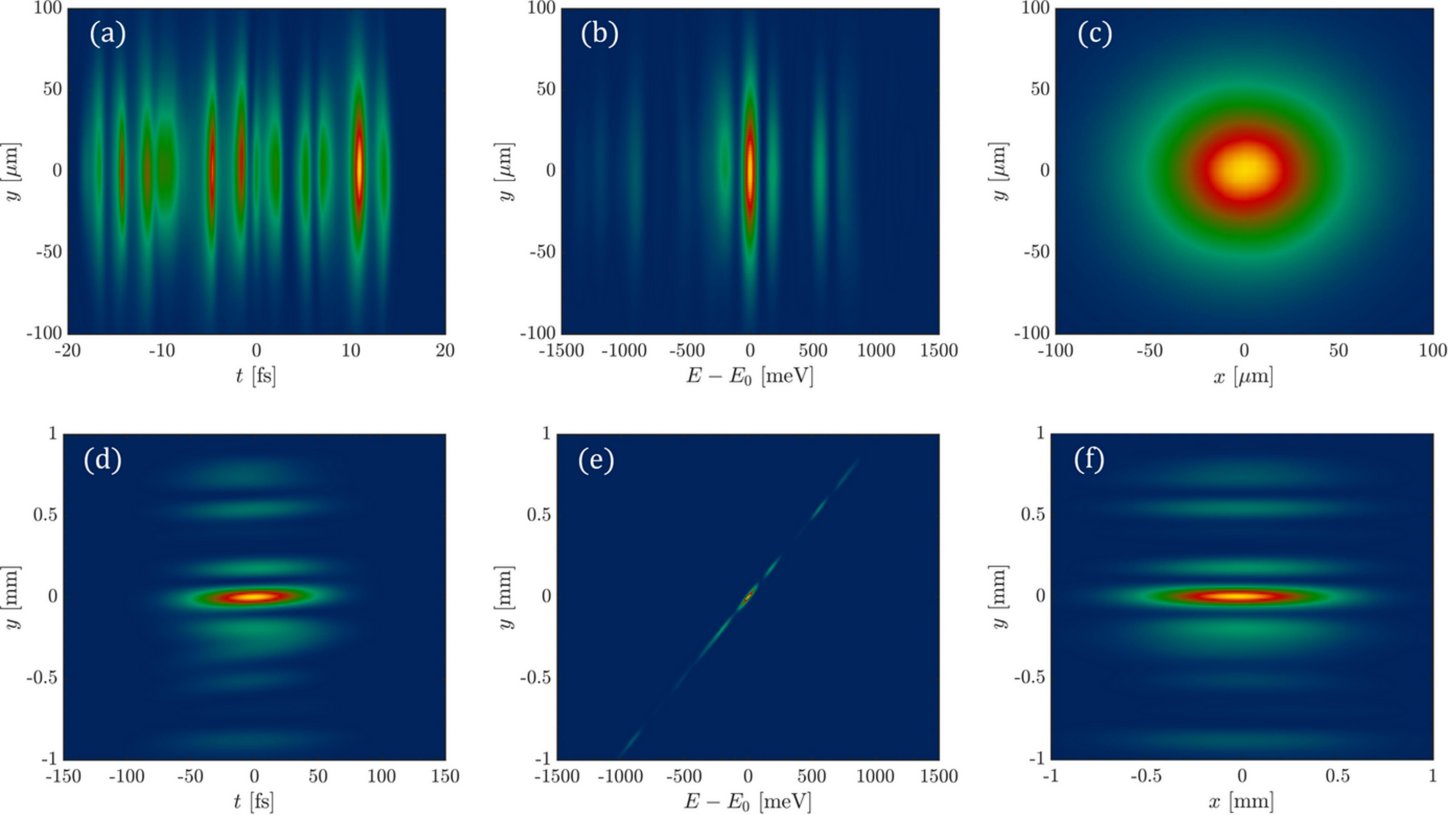
The properties of the FEL pulse at the source are displayed in (*a*) to (*c*). The light field information of the FEL pulse before the exit slit is illustrated in (*d*) to (*f*). (*a*, *d*) Intensity distribution in the (*y*, *t*) domain. (*b*, *e*) Intensity distribution in the (*y*, *E*) domain. (*c*, *f*) Intensity distribution in the (*y*, *x*) domain.

The 3-D FEL pulse is produced using *Genesis 1.3* (Reiche, 1999 ▸) and the central photon energy is 1240 eV. Using the 3-D FEL pulse propagation method in *FURION*, we can obtain the pulse intensity distribution of the 3-D FEL pulse at the grating image plane (118 m downstream of the grating). The properties of the FEL pulse at the source and before the exit slit are presented in Fig. 2 ▸. Figs. 2 ▸(*a*), 2(*b*) and 2(*c*) show the intensity distribution of the source in the (*y*, *t*), (*y*, *E*) and (*y*, *x*) domain, respectively. Multiple longitudinal modes are observed in the (*y*, *t*) domain, several spikes appear in the frequency (photon energy) domain and a near Gaussian distribution is evident in the (*y*, *x*) domain. Fig. 2 ▸(*d*) shows the intensity distribution at the image plane in the (*y*, *t*) domain, and we can observe that the intensity distribution in the *y* dimension precisely corresponds to the spectral distribution. Fig. 2 ▸(*e*) shows the intensity distribution at the image plane in the (*y*, *E*) domain in which the central photon energy *E*_0_ is 1240 eV. Fig. 2 ▸(*f*) shows the (*y*, *x*) domain distribution at the image plane, where several spikes are observed due to the linear distribution of photon energy along the *y* dimension.

**Figure 3:**
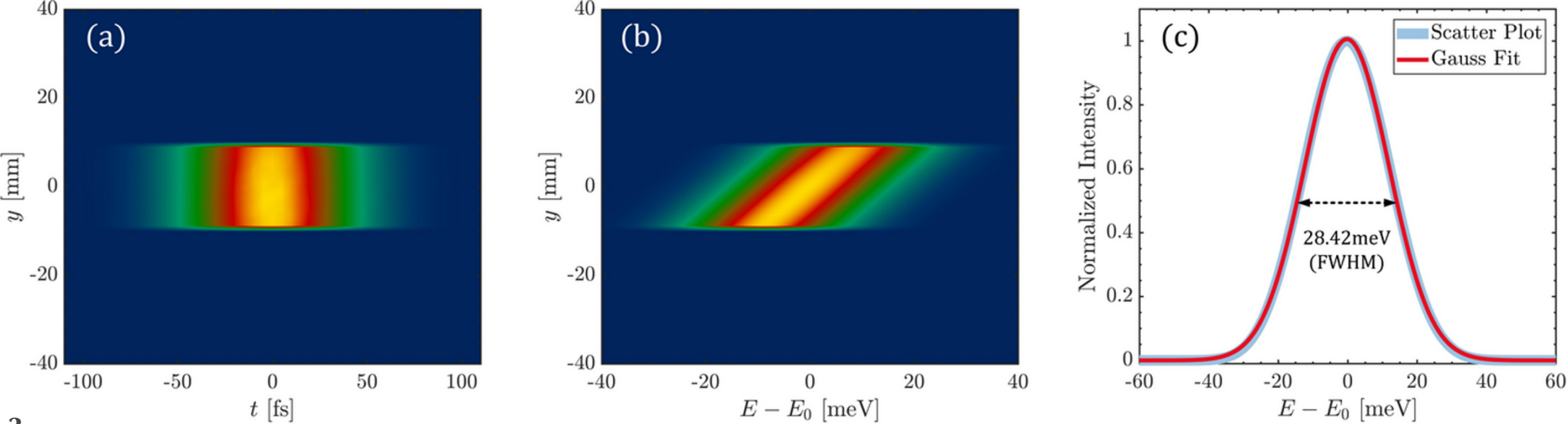
Properties of the pulse intensity distribution after the exit slit. (*a*) Intensity distribution in the (*y*, *t*) domain. (*b*) Intensity distribution in the (*y*, *E*) domain. (*c*) Spectrum after the slit, integrated along the *y* dimension.

At the location 118 m downstream of the grating (the image plane), the exit slit with a width of 20 µm is placed. Since the spectrum at the grating’s image plane is already distributed along the *y* dimension, the slit can be used to purify the spectrum. The FEL pulse intensity distribution after the slit is shown in Fig. 3 ▸. Fig. 3 ▸(*a*) depicts the intensity distribution in the (*y*, *t*) domain after the slit, which corresponds to the selected photons. By applying a slit along the *y* dimension in Fig. 2 ▸(*e*), we can obtain the intensity distribution after the slit in the (*y*, *E*) domain, as shown in Fig. 3 ▸(*b*).

To assess the resolving power of the monochromator, we integrated the spectrum obtained after the slit along the *y* dimension, producing a spectrum distribution corresponding to the blue curve in Fig. 3 ▸(*c*). Fitting a Gaussian function to this distribution yields a FWHM of 28.42 meV. This result demonstrates that the relationship between intensity and photon energy follows a Gaussian distribution. The FWHM of this post-slit spectrum, denoted as Δ*E*, represents the effective spectral bandwidth provided by the monochromator. By using *E*/Δ*E*, we determine that the theoretical resolving power of the grating monochromator at 1240 eV is 43630.

## 4. Impact of longitudinal source jitter on resolving power

The longitudinal source jitter affects the resolving power of the monochromator in two primary ways. Firstly, the jitter of the source position will alter the object distance of the VLS grating, which affects the geometric magnification. This change leads to a variation in the waist size of the focal spot at the image plane. Generally, when the source moves upstream, the waist size decreases, enhancing the resolving power. Conversely, moving downstream increases the waist size and reduces the resolving power. Secondly, the movement of the source results in a change of the image distance, thereby misaligning the slit position from the beam waist. This factor results in a reduction of resolving power regardless of whether the source shifts upstream or downstream. Consequently, when the source moves downstream, a decrease in resolving power is inevitable. However, when the source shifts upstream, both effects must be taken into account to fully understand their combined impact on resolving power.

**Figure 4:**
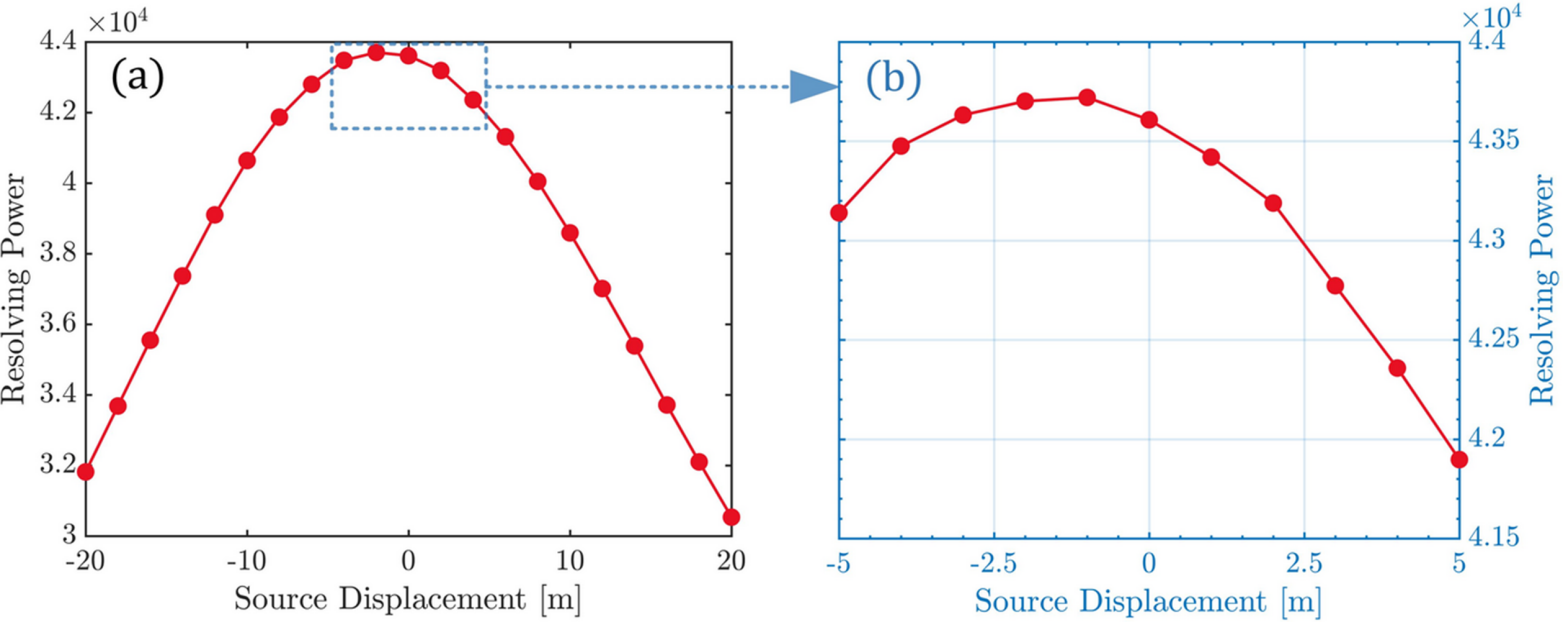
Impact of longitudinal source displacement on resolving power. (*a*) Source point movement of ±20 m. (*b*) Source point movement of ±5 m.

Fig. 4 ▸(*a*) illustrates the impact of a longitudinal source displacement of ±20 m on the grating’s resolving power. The figure shows that the second effect mentioned above predominantly influences the resolving power, resulting in a decrease in resolving power by more than 26% regardless of whether the source moves 20 m upstream or downstream. We specially investigate the change in resolving power when the source moves longitudinally by ±5 m (equivalent to the length of one undulator section), as depicted in Fig. 4 ▸(*b*). It can be observed that, when the source moves a short distance upstream (less than 3 m), the first effect dominates, causing an increase in resolving power rather than a decrease. However, when the source moves more than 3 m upstream, the second effect becomes predominant, leading to a rapid decline in resolving power. The figure shows that shifting the source 5 m upstream results in an approximately 1% decrease in resolving power, while moving it 5 m downstream leads to about a 4% decrease.

## 5. Impact of grating aperture on resolving power

The fabrication of long, high-precision gratings is challenging and a longer grating also implies higher costs. Therefore, an effective method for evaluating the impact of grating size on optical systems is necessary. Resolving power is one of the most important characteristics of a grating monochromator, making it reasonable to use the impact of grating size on resolving power as an evaluation criterion (Gerasimova *et al.*, 2022 ▸; Nicolas & Cocco, 2022 ▸).

**Figure 5:**
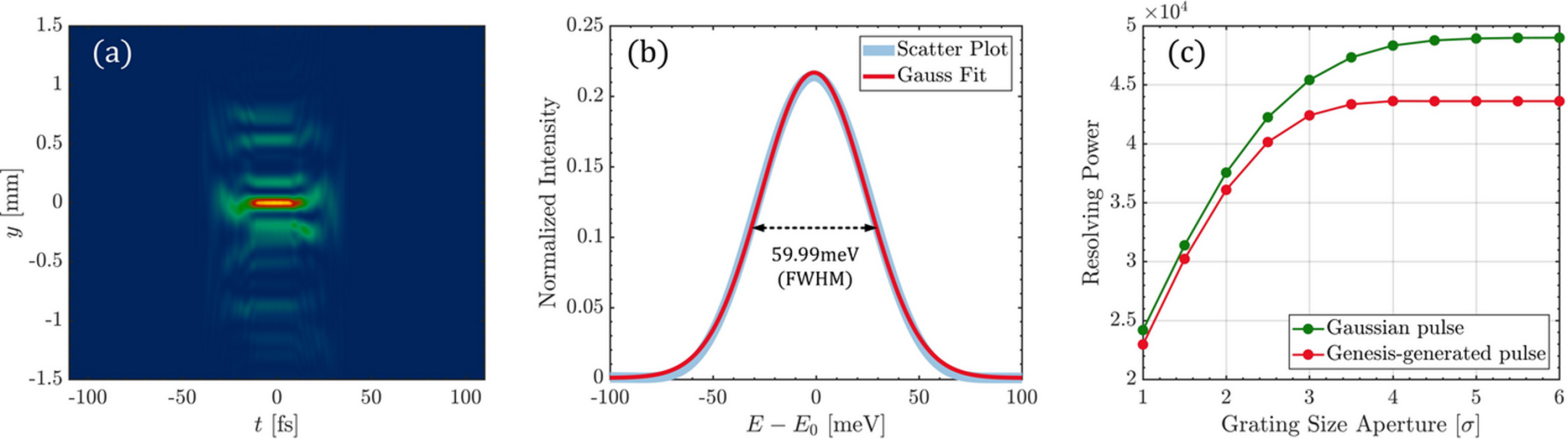
Impact of a 50 mm grating size on resolving power. (*a*) Wavefront distortion occurs in the (*y*, *t*) domain before the slit. (*b*) The aperture diffraction effect of the grating causes spectral broadening of the integrated spectrum obtained after the slit. (*c*) Impact of the grating aperture on the monochromator’s resolving power.

As the grating size decreases, the diffraction effects become more pronounced, leading to a greater impact of the grating’s aperture effect on resolving power. In numerical simulations, the effect of grating size can be approximated by adding a vertical slit in the *y* dimension immediately downstream of the grating with a size equivalent to the grating’s projected size. The aperture size *A* formed by the grating size *L* can be expressed as 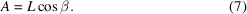
For the case discussed in this paper, setting the grating size to 50 mm reveals a significant impact of the grating aperture effect. Specifically, noticeable wavefront distortion occurs in the (*y*, *t*) domain in front of the slit, as shown in Fig. 5 ▸(*a*). Additionally, the spectrum integrated along the *y* dimension behind the slit is broadened due to the grating aperture effect, resulting in a reduction of the monochromator’s resolving power to approximately 20670, as depicted in Fig. 5 ▸(*b*).

To select an appropriate grating size, we evaluated the impact of different grating sizes on resolving power. The FEL pulse footprint on the grating approximates a Gaussian profile, with σ denoting the root-mean-square (RMS) footprint size along the grating’s length direction. In the numerical simulations, various grating sizes corresponding to 1σ to 6σ of the footprint size are assessed using pulse propagation methods to evaluate their impact on resolving power. In Fig. 5 ▸(*c*), the red dotted line represents the resolving power versus grating size obtained from simulations using *Genesis*-generated pulses. This result indicates that, for the FEL-1 monochromator at 1240 eV, the aperture diffraction effect of the grating becomes negligible when the grating covers larger than 4σ beam size, corresponding to a grating size of 236 mm. For comparison, the green dotted line represents the results obtained using a Gaussian beam of the same spot size and bandwidth, which exhibits higher resolving power.

## 6. Impact of thermal deformation on resolving power

**Figure 6:**
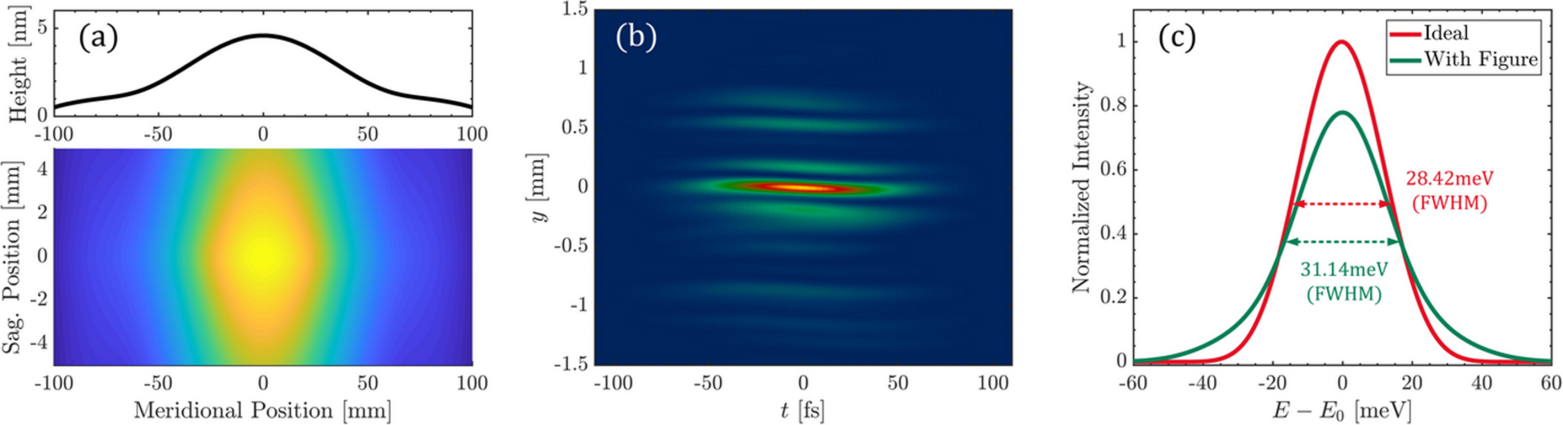
Impact of a 1.38 nm (RMS) thermal deformation on resolving power. The absorption power of the grating was 2.14 W @ 1240 eV. The simulation was conducted at a repetition rate of 100 kHz with an energy per pulse of 200 µJ. (*a*) Thermal deformation distribution and meridional height error of the grating. (*b*) Wavefront distortion occurs in the (*y*, *t*) domain before the slit. (*c*) The thermal deformation of the grating causes spectral broadening of the integrated spectrum obtained after the slit.

The thermal load in high-repetition-rate FELs imposes significant impacts on the performance of optical components. For grating monochromators, the resolving power serves as a critical metric for evaluating the impact of thermal surface deformations. For the *i*th pulse slice in the (*y*, *t*) plane, the thermal deformation-induced wavefront perturbation can be modeled as an additional phase term, 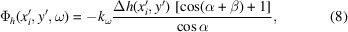
where Δ*h*(*x*_*i*_′, *y*′) denotes the height error for the corresponding position on the exit plane (*x*_*i*_′, *y*′). Incorporating this phase term into equation (3) yields the modified expression 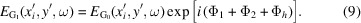
Taking the optimized grating surface at a repetition rate of 100 kHz as an example, the calculated output power per pulse is 200 µJ at 1240 eV. Using the CXRO website (Henke *et al.*, 1993 ▸), the reflectivities of M1, M2 and M3 were calculated to be 95.23%, 95.23% and 89.99%, respectively. The grating’s diffraction efficiency was computed using the *Reflec* code (Schafers & Krumrey, 1996 ▸). After accounting for all diffraction orders, the grating’s absorption rate was determined to be 13.11%, corresponding to an absorption power of 2.14 W. The effective grating length is 200 mm. Fig. 6 ▸(*a*) presents the optimized thermal deformation profile of the water-cooled grating obtained through finite element analysis. The figure highlights the height error along the meridional centerline, clearly revealing a thermal bump with a height error of 1.38 nm (RMS). Fig. 6 ▸(*b*) illustrates the intensity distribution in the (*y*, *t*) domain before the exit slit, revealing a slight tilt due to thermally induced image plane displacement. Fig. 6 ▸(*c*) demonstrates a significant degradation in resolving power: the spectral FWHM broadens from 28.42 meV to 31.14 meV, with a corresponding resolving power decrease from 43630 to 39820 (8.7% reduction). Notably, although the current thermal impact remains acceptable, increasing the repetition rate to 1 MHz may amplify the thermal deformation by an order of magnitude, potentially resulting in unacceptable performance degradation. This underscores the urgent need for advanced thermal management strategies in MHz-class high-repetition-rate FEL systems.

## 7. Summary

This paper primarily discusses how to evaluate the resolving power of a pulsed FEL passing through a grating monochromator using the pulse propagation method. Three applications use resolving power as the evaluation criterion. The first one involves quantitatively analyzing the impact of longitudinal displacement of the FEL source on resolving power. The second one involves assessing the aperture diffraction effect caused by grating size, providing guidance for selecting the appropriate grating size. The third one involves evaluating the influence of thermal deformation on the monochromator’s performance.

Using our previously developed *FURION* software, the start-to-end FEL pulse propagation can be simulated. By analyzing the spectrum at the monochromator’s exit slit, we can determine its resolving power. For the FEL-1 SCI experimental endstation at the photon energy of 1240 eV, a longitudinal position shift of the source by one undulator period (±5 m) results in a maximum decrease in resolving power of 4%. The evaluation of grating size shows that the aperture diffraction effect of the grating becomes negligible when the grating size covers 4σ beam size. Simulations of grating thermal deformation at 100 kHz indicate that an RMS height error of 1.38 nm can result in an 8.7% reduction in resolving power.

This work introduces a more realistic numerical assessment of the performance of a grating monochromator and provides valuable guidance for the design and optimization of FEL beamlines.

## Figures and Tables

**Table 1:** Grating parameters used for simulation

Parameter	Value	Unit		Parameter	Value	Unit
*n*_0_	300	lines mm^−1^		*C*_ff_	1.8	–
*r*_1_	207217	m		*r*_2_	118108	m
α	89.062	°		β	88.312	°
*b*_2_	0.02884	m^−1^				
